# Informal carers’ perspectives on the delivery of acute hospital care for patients with dementia: a systematic review

**DOI:** 10.1186/s12877-018-0710-x

**Published:** 2018-01-25

**Authors:** Sarah Beardon, Kiran Patel, Bethan Davies, Helen Ward

**Affiliations:** Patient Experience Research Centre, Imperial College London, Medical School Building, St Mary’s Campus, Norfolk Place, London, W2 1PG UK

**Keywords:** Dementia, Carers, Hospital, Experience, Qualitative

## Abstract

**Background:**

Providing high quality acute hospital care for patients with dementia is an increasing challenge as the prevalence of the disease rises. Informal carers of people with dementia are a critical resource for improving inpatient care, due to their insights into patients’ needs and preferences. We summarise informal carers’ perspectives of acute hospital care to inform best practice service delivery.

**Methods:**

We conducted a systematic search of bibliographic databases and sought relevant grey literature. We used thematic synthesis analysis to assimilate results of the studies and describe components of care that influence perceived quality.

**Results:**

Twenty papers met the inclusion criteria. Findings identified four overarching components of care that influenced carer experience and their perceptions of care quality: ‘Patient care’, ‘Staff interactions’, ‘Carer’s situation’ and ‘Hospital environment’. Need for improvement was identified in staff training, provision of help with personal care needs, and dignified treatment of patients. Carers need to be informed, involved and supported during hospital admission in order to promote the most positive experience.

**Conclusion:**

This review identifies common perspectives of informal carers of people with dementia in the acute hospital setting and highlights important areas to address to improve the experience of an admission for both carer and patient.

## Background

In the UK, the quality of hospital care for patients with dementia has been widely criticised and attention is focused on achieving improvement [1, 2]. Guidelines, assessment tools and incentives have been developed to promote dementia friendly hospital environments, including the National Dementia Strategy, a Commissioning for Quality and Innovation (CQIN) reporting framework, inspection by the Care Quality Commission (CQC) and the National Audit of Dementia [3–7].

Globally, the rising number of people living with dementia is creating significant challenges to the provision of appropriate care in the community and hospital [8]. In England, people over 65 years contribute about two thirds of hospital bed days [9]. The estimated population-level prevalence of dementia in this age group is 5% [10, 11]. However, amongst hospital inpatients, the prevalence of dementia is likely to be markedly higher. The UK Royal College of Psychiatrists estimate that a “mental disorder” will be diagnosed in up to 60% of hospital admission in people over 65 [9]. A systematic review found that in high-income settings, the prevalence of dementia in inpatients on medical wards aged over 65 years ranged from 9.1% to 40.0% and in people over 70, the prevalence ranged from 35.2% to 43.2% [12]. There is uncertainty in these estimates due to the marked heterogeneity between settings.

Maintaining independence and managing daily activities becomes challenging for people with dementia as they become increasingly dependent on care provided by other people. A large proportion of this care is provided by informal carers, usually family members (including spouses, children, siblings) [8, 13]. Informal care is defined as the provision of support to sick, elderly or disabled people in a non-professional capacity, usually unremunerated and unchosen [14]. It is widely recognised that caring for a person with dementia can cause significant strain to carers, including psychological distress, poor physical health, social isolation, poor quality of life, financial burden and grief [13, 15–19]. Hospital admissions can also exacerbate carer stress and vulnerability [20].

Informal carers can provide unique insights into the needs and preferences of patients with dementia. By interacting with the healthcare system they can mediate and advocate on behalf of the patient, thereby also supporting the work of healthcare professionals to provide the most appropriate care [3, 21]. The key role that carers can play in improving inpatient care is well recognised [22].

Person-centred care (PCC) is a key concept in the theory of care for people with dementia. This best-practice approach recognises that the wellbeing of the person with dementia is enhanced if carers are able to support their personhood through a social interdependence [23]. To support the application of a PCC model into practice it has been translated into four key elements known as the VIPS framework: (1) Valuing people with dementia and those who care for them; (2) treating people as Individuals; (3) looking at the world from the Perspective of the person with dementia; and (4) a positive Social environment in which the person living with dementia can experience relative wellbeing [24]. The VIPS framework has been adapted for use in nursing homes [25] and in the ‘hospital setting’ the PCC theory is supported by the “Triangle of Care” model that recognises an equal partnership between the person with dementia, healthcare practitioners and informal carers [26].

While the healthcare experience of people with dementia and their carers has been reviewed in primary care settings, there has been no review of the evidence on informal carers’ perspectives on the delivery of acute hospital care for patients with dementia [27]. We therefore conducted a systematic review to assemble and synthesize the published research evidence to describe common perspectives and experiences of informal carers of patients with dementia during acute hospital admission [28]. This information is needed to inform best practice delivery of acute hospital care for patients with dementia.

## Methods

### Systematic review

The following databases were searched by two reviewers: Medline, Embase, Health Management Information Consortium, and PsycINFO. Using best practice guidelines, search strategies were developed for each database [29] (See Appendix 1 for MEDLINE search strategy). The search was broadened through scanning references and the publication lists of key authors. Grey literature was sought through Google, Google Scholar and ResearchGate, using a combination of free text search terms relating to dementia, acute hospital services, carers, care quality and experiences. The websites of relevant organisations were also searched for publications (See Appendix 2).

Elements of the research question were defined using the SPIDER search tool for qualitative and mixed methods studies [30]. Eligibility criteria were: 1) Sample: informal carers of people who have dementia; 2) Phenomenon of interest: delivery of acute hospital care; 3) Design: studies collecting primary data from carers; 4) Evaluation: experiences and perceptions of care; 5) Research type: qualitative (interviews or focus groups) or quantitative (surveys). Studies with no full text available or non-English language were excluded. There were no exclusions by date. The search and study selection were performed according to the PRISMA statement guidelines and carried out by two researchers (SB and KP).

Relevant findings from the studies were extracted, including data from surveys, quotes from interviews and descriptions or summaries of findings from qualitative research. The quality of evidence provided by each paper was assessed using criteria based on the Critical Appraisal Skills Programme (CASP) for evaluation of qualitative research; the criteria were adapted to also apply to quantitative and mixed methods studies, as set out in Table 1 [31–33]. Papers were graded as high, moderate or low quality based on the total of their ratings for each criterion; grading was carried out by two researchers independently (SB and KP) and their ratings were compared to agree the final allocation. Quality rating did not affect whether publications were included.

**Table 1:** Adapted CASP qualitative research checklist

Are the research questions clearly stated?
Is the setting and context clearly described?
Is there an appropriate, well-described sampling strategy?
Is there an appropriate, well-described data collection strategy?
Is there an appropriate, well-described data analysis method?
Are the claims made supported by sufficient evidence?
Is the role and reflexivity of the researcher adequately described?

### Narrative thematic synthesis

A narrative thematic synthesis approach was chosen for the analysis of studies’ results. This method provides an effective way of synthesising qualitative information, which was the predominant methodology of included studies [34]; however, it can also be applied to the quantitative and mixed methods studies by classifying the themes in the results, and therefore allowed the findings of all studies to be compared based on their original context.

The three steps of the thematic synthesis method were used [34]: 1) Coding findings - material was coded line-by-line to identify initial motifs emerging in the data; 2) Developing descriptive themes - initial codes (motifs) were expanded into broader themes by comparison and translation across the studies; 3) Developing analytical themes - key themes and sub-themes were established through further conceptualisation of the material, discussion and analysis. This analysis was carried out by two researchers independently (SB and KP), the findings were combined by SB and the results reviewed by all authors.

## Results

**Fig. 1:**
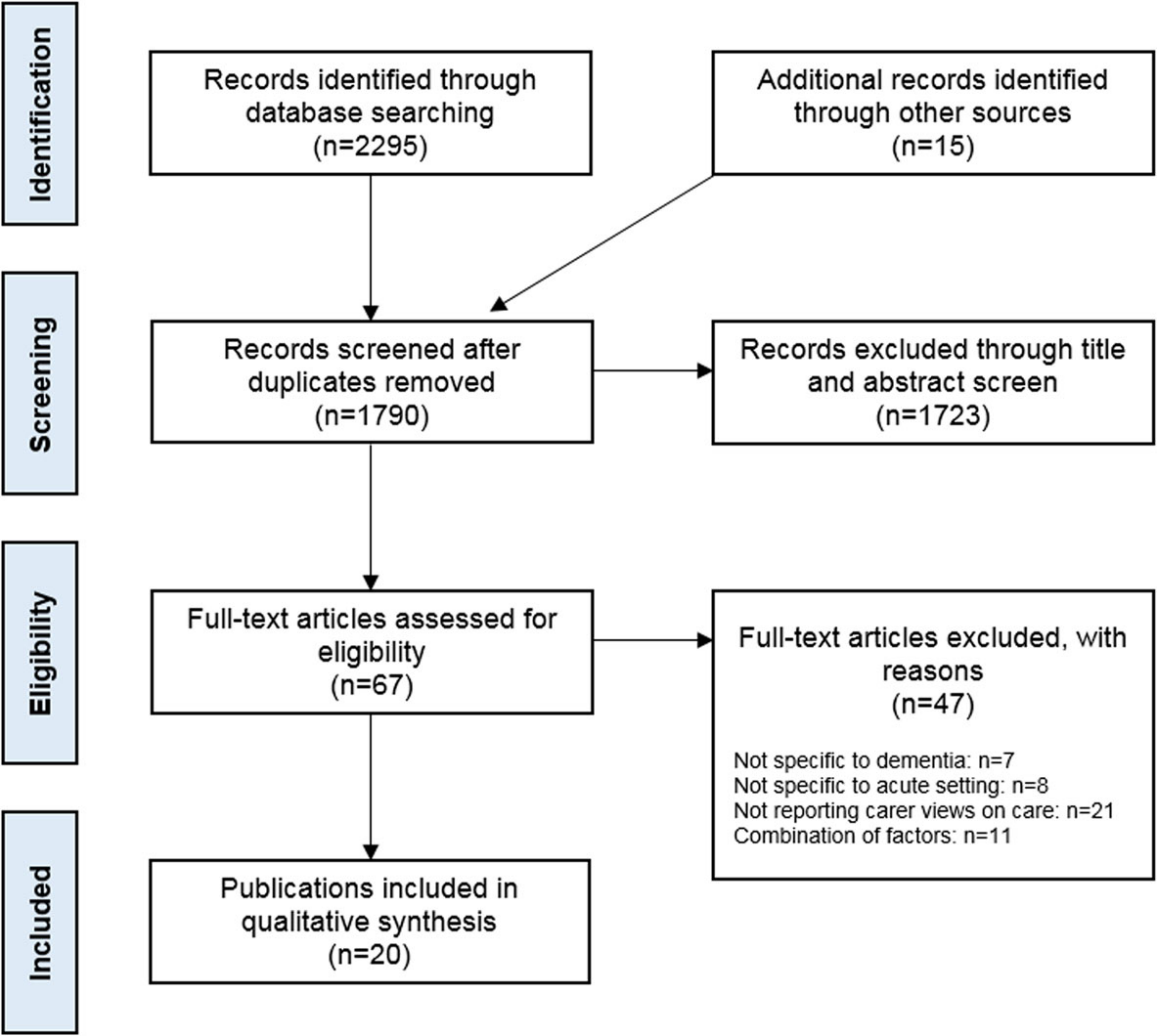
PRISMA flow diagram

The systematic search and selection process resulted in 20 publications included in the analysis [2, 20, 35–52] (Fig. 1). Study characteristics and quality assessments are shown in Table 2. Two of the included papers originated from Australia, the remaining 18 from the UK. The majority of included papers were research articles (*n* = 16), followed by doctoral theses (*n* = 2), public programme reports (*n* = 1) and charity reports (*n* = 1). All of the papers reported carer feedback. Sixteen papers used qualitative approaches for data collection (interviewing and observation), three were quantitative analyses of surveys and one used mixed methods. The quality of research was variable, with a mixture of high (*n* = 8), moderate (*n* = 6) and low (*n* = 6) quality papers. In total, this review encompasses the views of 1993 carers, 189 of whom took part in qualitative interviews, 1763 who responded to surveys and 41 who did both.

**Table 2:** Study characteristics

Authors	Country	Setting	Participants	Data collection method	Data analysis method	Publication type	Study quality	Contribution to synthesis
Clissett et al. (2013)	UK	11 wards (general medical, healthcare for older people and orthopaedic) in 2 acute hospitals	29 carers	Non-participant observation and interviews	Thematic analysis	Research article	High	Medium
Clissett et al. (2013)	UK	12 wards (general medical, healthcare for older people and orthopaedic) in 2 acute hospitals	34 carers	Non-participant observation and interviews	Constant comparison analysis	Research article	High	Large
Clissett et al. (2014)	UK	12 wards (general medical, healthcare for older people and orthopaedic) in 2 acute hospitals	35 carers	Non-participant observation and interviews	Constant comparison analysis	Research article	High	Small
Cowdell (2007)	UK	3 wards in a specialist older people’s unit in an acute hospital	7 carers	Participant observation, informal conversations and interviews	Critical interpretive ethnography and narrative analysis	Doctoral thesis	Moderate	Medium
Dening et al. (2012)	UK	Health and social care system of a London borough (Haringey)	7 carers	Semi-structured interviews	Thematic analysis	Research article	Moderate	Small
Douglas-Dunbar and Gardiner (2007)	UK	District general hospital	9 carers	Interview	Not specified	Research article	Low	Medium
Gladman et al. (2012)	UK	11 wards (general medical, healthcare for older people and orthopaedic) in 2 acute hospitals	35 carers	Non-participant observation and interviews	Constant comparison analysis	Public programme report	High	Large
Gonski and Moon (2012)	Australia	10-bed secure unit providing intensive nursing care	10 carers	Questionnaires	Quantitative and thematic analysis	Research article	Low	Small
Jamieson et al. (2014)	Australia	Community	30 carers	Telephone interviews	Thematic analysis	Research article	Moderate	Medium
Jurgens et al. (2015)	UK	12 wards (general medical, healthcare for older people and orthopaedic) in 2 acute hospitals	35 carers	Interviews	Constant comparison analysis	Research article	High	Large
Lakey (2009)	UK	Hospitals across the country	1291 responses, 96% from carers	Questionnaire	Not specified	Charity report	Low	Medium
Porock et al. (2015)	UK	11 wards (general medical, healthcare for older people and orthopaedic) in 2 acute hospitals	35 carers	Non-participant observation and interviews	Constant comparison analysis	Research article	High	Medium
Simpson (2016)	UK	Local carers’ support groups and a day centre	7 carers	Semi-structured interviews	Thematic analysis	Research article	Low	Medium
Simpson et al. (1995)	UK	7 inpatient wards within a Mental Health Service Trust	41 carers	Interviews and questionnaires	Grounded theory and descriptive statistics	Research article	Moderate	Medium
Spencer et al. (2013)	UK	Specialist medical and mental health unit and standard care wards	40 carers	Semi-structured interviews	Thematic analysis	Research article	High	Large
Taylor (1998)	Australia	Not specified	20 carers	Semi-structured interviews	Thematic analysis	Research article	Low	Small
Telford (2015)	UK	Hospitals across the country	8 carers	Semi-structured interviews	Constructivist grounded theory analysis	Doctoral thesis	Moderate	Large
Thune-Boyle (2010)	UK	1 acute hospital	20 carers	Semi-structured interviews	Thematic analysis	Research article	Moderate	Small
Tolson et al. (1999)	UK	Not specified	41 patients and main visitors	Critical incident interviews	Conceptual triangulation	Research article	Low	Small
Whittamore et al.	UK	One general hospital	462 carers	Questionnaires	Cohort analysis and descriptive statistics	Research article	High	Medium

A large contribution to the research in this area has come from one large randomised controlled trial (RCT) conducted in a single NHS Trust in England. The findings from this work therefore significantly influence the overall synthesis presented in this review. Seven of the papers from this RCT report results from 29 to 40 interviews with carers and 72 h of observation on hospital wards [37, 40, 43, 44, 50–52]. One further paper reports the results of a questionnaire study with 462 respondents conducted as part of the trial [45].

**Fig. 2:**
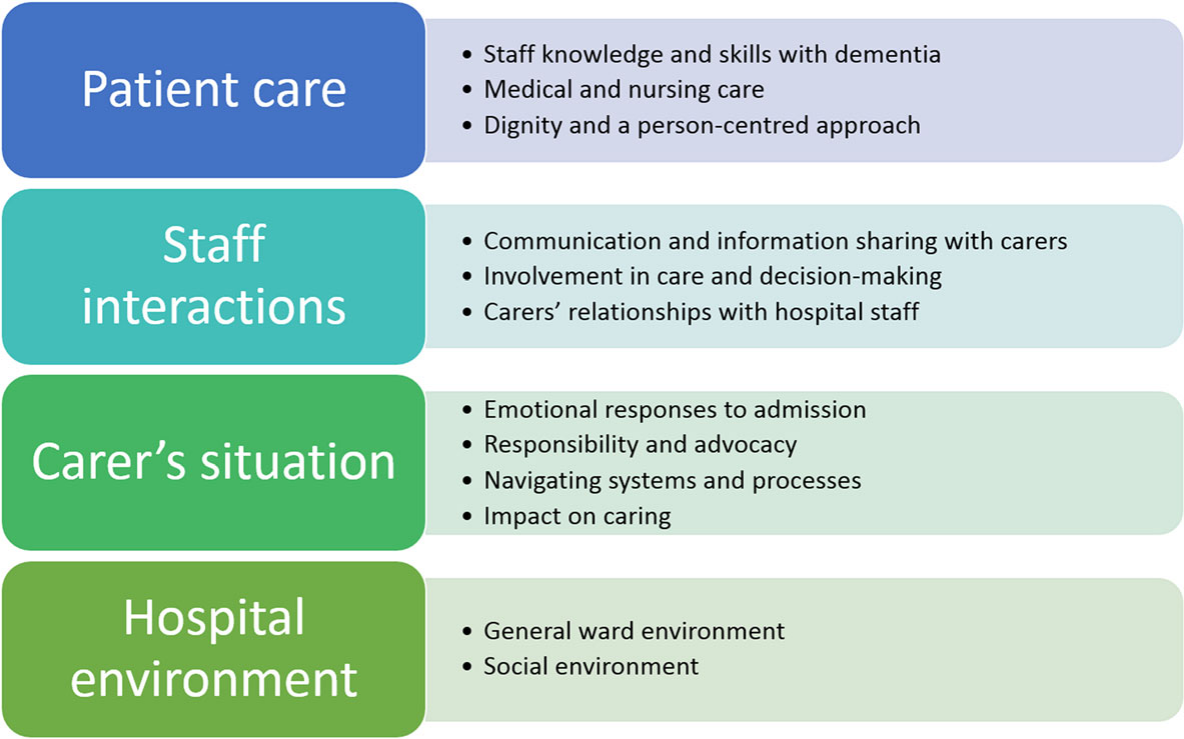
Theoretical framework representing overall results of the review

We identified four overarching themes within the carers’ perspectives on the delivery of acute hospital care for patients with dementia: ‘Patient care’, ‘Staff interactions’, ‘Carer’s situation’ and ‘Hospital environment’ (Fig. 2).

### Patient care

#### Staff knowledge and skills with dementia

Study findings repeatedly highlighted carers’ concerns that hospital staff did not understand dementia, were untrained and unskilled in managing patients with dementia and could not provide for the needs of this patient group [2, 20, 36, 37, 39–43, 46, 52]. The prevalence of this concern was demonstrated by a survey showing that 67% of carers were dissatisfied with staff who they felt did not recognise cognitive impairment [2]. Staff were perceived not to recognise the specific needs and vulnerabilities of patients with dementia [41, 46] and sometimes to lack patience [40]. This perceived lack of professional knowledge led carers to worry for the safety of the patient, as staff might assume they could recall facts and make decisions [38]. In a study of a specialist medical and mental health unit within an acute hospital, carers gave positive reports of staff being well prepared and displaying positive attitudes towards managing patients with dementia [40].

#### Medical and nursing care

Some studies highlighted that carers were usually contented with the general care provided in hospital and appreciated the efforts of staff [20, 47–49]. However, one survey found that the management of medical problems was one of the most common causes of discontent (22% of carers dissatisfied with this aspect of care), and was an important determinant of overall sentiment [45].

A frequent complaint was the failure of the hospital to provide for basic personal care needs, including help with washing, dressing, toileting, eating, drinking or taking medication [2, 36, 40, 42, 43, 46]. Carers reported appreciation when they witnessed staff proactively helping the patient with these aspects of care [40, 42, 50]. Sensitive provision of personal and intimate care was an important component of perceived good practice, with lack of attention to cleanliness and dignity causing great concern for carers [40].

Omissions in basic care provision were partly attributed to a lack of understanding and compassion on the part of the staff, although low staffing levels were also cited [36]. Carers sympathised with nursing staff being overstretched and acknowledged the strain on the system, despite witnessing inadequacies in care [20, 43, 44, 46]. However, some reported frustration at slow response to call bells or having to ask repeatedly for nurses to attend the patient [46, 50].

#### Dignity and a person-centred approach

A perceived lack of dignity and respect towards patients with dementia was cited as a concern for carers in several studies [2, 36, 40, 48], including a large survey that found 36% of carers reporting that patients were never treated with dignity and respect [2]. Carers described distress at witnessing hospital staff treating confused patients with a lack of understanding and compassion [36].

Some carers held the view that hospital care was task-focussed and medically oriented, not delivering treatment in a way that took individual needs and preferences into account [20, 40, 41, 44]. In one survey, 68% said hospital care was not person-centred [2]. This was partly attributed to how busy staff on the ward appeared to be, and to competing demands of the system, which were seen to impact negatively on the quality of care delivery and hinder a person-centred approach [20, 41, 42, 52].

Carer feedback highlighted the importance of staff displaying warm, positive attitudes and building caring relationships with patients in order to preserve identity and provide a person-centred experience [50]. Carers recognised and appreciated nurses taking trouble with patients beyond that of providing essential care, as evidenced by staff being positive and welcoming towards patients and demonstrating flexibility and helpfulness in caring for individuals [46].

### Staff interactions

#### Communication and information sharing with carers

Studies consistently highlighted the need for good communication between hospital staff and carers, with poor communication and information provision frequently cited as a grievance [40, 41, 45, 46, 48, 52]. Carers wanted regular communication [35, 40] and frequently needed more information than they received, including details about the patient’s medical condition, disease progression and symptoms, treatment options and care plans [39, 44, 52]. Carers described being left uninformed unless they questioned staff themselves [40] and had to seek information not knowing whom to approach [44, 48]. While one study reported carers actively pushing for information [44], others described them feeling reluctant to interrupt or disturb hospital staff despite wanting to know more [40, 48]. Communication was found to be poor throughout the hospital stay, from admission to discharge [20, 40].

One survey of carers [45] found that 34% were unhappy with how well they were kept informed; in this study it was the greatest cause for dissatisfaction with hospital care as well as being significantly associated with overall rating, demonstrating its strong influence on carers’ views [45]. This finding was confirmed by another study showing that poor communication led carers to express greater dissatisfaction with care quality [40]. Similarly, good communication improved carers’ satisfaction and trust in hospital care [37] and positive feedback highlighted informative staff [40]. Poor communication may act to cause dissatisfaction by leading carers to feel out-of-control and uncertain if adequate care is being provided [44].

#### Involvement in care and decision-making

Many studies reported that carers felt excluded from the hospital decision-making process and not listened to when they provided information about the patient and their needs [20, 37, 47–49]. Lack of involvement led to feelings of powerlessness, dissatisfaction and frustration. In a large survey of over 1000 carers, 43% reported not being involved in decision-making as much as they had wanted [2]. Effective engagement of carers during the hospital admission was found to be necessary to build good relationships and promote contentment with care [52].

Carers wanted to use their knowledge of the patient to influence care and ensure their needs were met [20, 44, 49]. However, staff often failed to seek carer input or to use their expertise in developing care plans, which led to frustration and resentment [20, 46, 48, 49, 52]. Some carers reported finding it difficult to approach medical staff to discuss their concerns or volunteer information [47, 52]. Despite often wanting to be involved in decision-making, some carers did not feel willing or capable to make decisions about medical treatment options and wished to defer final responsibility [52].

There was variation in carers’ feelings towards being directly involved in caring for the patient while in hospital [41, 49, 52]. Some carers wanted to provide hands-on care for the patient as they were used to doing, in order to maintain a sense of normality and to reassure the patient, as well as to express gratitude towards nurses [41, 44, 52]. In these cases, sometimes staff prevented them from doing so [52]. Others did not want to be directly engaged, or felt pressured into doing so to fill gaps in care [38, 40, 41, 52].

#### Carers’ relationships with hospital staff

Staff attitude towards carers was found to have a strong influence on carer satisfaction with the service and confidence in the quality of care [48]. Studies described “defensive”, “confrontational” and “patronising” attitudes of healthcare professionals, as well as carers feeling deliberately ignored by them [43, 52]. Other studies found that carers felt their concerns were not taken seriously and information they provided about patient needs was often disregarded [20, 37, 38, 48, 49].

Carers who described poor quality relationships were more likely to be discontented with services, particularly when staff failed to engage positively with them, actively disregarded family input or were not perceived to attempt to build connections [37, 40, 51]. Similarly, warm relationships increased satisfaction and trust in the service, with carers feeling reassured when staff recognised the importance of their relationship with the patient and involved them appropriately in care [37, 51].

### Carer’s situation

#### Emotional responses to admission

Carers experienced significant worry about the patient’s wellbeing in hospital. This included distress caused by the acute illness, as well as concern about their mental state and the potential for deterioration during a hospital admission [20, 35, 37, 38, 43, 46, 52]. Due to patients’ communication difficulties, carers worried that they might not be able to make themselves understood if they were frightened, hungry or in pain [37].

Carers described the stressfulness of the admission from their own point of view, feeling vulnerable in the unfamiliar hospital system [20, 35] as well as physically and emotionally exhausted [20, 52]. They described their need for understanding and emotional support from the staff, as well as a particular need for support with information-seeking [20].

#### Responsibility and advocacy

Carers commonly felt the need to advocate on behalf of patients to ensure their care needs were met in hospital [20, 41, 44, 46, 52]. This led to them spending long periods of time on the ward and continuing to provide physical and emotional care [38, 41]. Feelings of responsibility were due to a number of factors, including having personal insights into the needs of the patient and needing to communicate on their behalf [41]. However, it also included witnessing shortfalls in care and not trusting that proper care would be provided in their absence [41, 46]. Some described feeling obliged to be present and help provide care because staff requested them to [41, 46].

Some carers expressed considerable gratitude for the care received in hospital, and relief at having the responsibility of caring lifted from them [41, 48, 49]. However, one study reported that the inpatient experience was more stressful than daily life due to the hospital requesting them to be present to help manage the patient [38]. This was supported by findings from another study showing that admission did not provide respite for carers, but rather created stress due to extra travelling and disruption to their normal routine [52].

#### Navigating systems and processes

One of the most prominent areas of frustration for carers was the hospital discharge planning process: one survey found 29% of carers were dissatisfied with this aspect of the hospital experience [45], and another reported around half [47]. Dissatisfaction with discharge was strongly associated with overall rating of care, demonstrating its influence on carer experience [45]. Negative perspectives were described when discharge arrangements were poorly planned and made without consulting the carer [37, 38, 40, 41, 52]. Inappropriate or chaotic discharge planning was a major source of anxiety and frustration, causing carers to feel powerless and distrustful towards the hospital [37, 38, 41]. One study reported that carers felt visiting times were often inflexible and insufficient for their needs [41].

During the hospital stay, carers reported concern about the impact that the admission could have on existing care arrangements in the community, including worries that community support services would be withdrawn due to extended hospital stays [37, 43, 44]. Planning for care of the patient after discharge was another significant strain, with the possibility of long term residential placement becoming necessary causing considerable grief as well as financial concerns [49].

### The hospital environment

#### General ward environment

The general ward environment was perceived by carers to be unsuitable for patients with dementia, not being conducive to the management of distress and confusion and potentially contributing to deteriorating health of the patient: carers noted worsening mental state and behaviour, hospital acquired infections, bedsores and falls [36, 41, 44]. They expected the ward to be a place of safety where patients with behavioural disturbances could be appropriately managed, and expressed concern when they found this was not the case [52]. Incidents of actual or potential harm were reported [46].

Lack of cleanliness was sometimes highlighted by carers, as well as unattractive décor and impersonal surroundings [40]. Others described the ‘bleakness’ of the hospital environment as one of the worst aspects of the hospital stay [48]. Carers in one study found the hospital environment uncomfortable for visiting, lacking sufficient facilities such as chairs and refreshments [37]. Carers were also concerned about frequent ward moves [52], which contributed to stressful losses of personal possessions that sometimes left the impression of an inadequate service [42, 46].

#### Social environment

Carers reported mixed preferences about the social environment in hospital. Some were concerned about the lack of privacy on shared wards [40, 41], which was reported as one of the worst aspects of the admission by one carer survey [48]. Contrary to this, others described patients’ distress when they were isolated in a separate room [41]. Some carers were unhappy about the disruptive behaviour of other patients on the ward [37, 47, 48] or worried that their own relative would cause disturbance [35, 46].

A large survey found that 62% of carer respondents were dissatisfied with opportunities for social interaction for patients while in hospital [2]. This was corroborated by other studies in which carers reported insufficient or inappropriate provision of activities and opportunities for social engagement [40, 41]. They felt this would leave patients bored or cause behaviours like wandering and shouting [40], however they also recognised that many patients were too unwell to be willing or able to socialise [41]. Carers often took on this role of providing company and stimulation for patients [40, 41].

### Interactions between themes

The four themes identified in this review are related and can be seen to interact, reflecting the complexity in how different aspects of healthcare determine carer experience. For example, the standards of patient care witnessed could influence their opinions of staff and of the hospital system. The nature of staff interactions could also affect perceptions of patient care, hospital systems and the ward environment. The carer’s own concerns about the person with dementia were influenced by the care they observed in hospital and the quality of communication with staff, which could in turn affect the way carers interacted with staff and how satisfied they were with the care provided.

## Discussion

### Principal findings

This review describes the perceptions of informal carers of people with dementia who receive care during an acute hospital admission. We present four themes ‘Patient care’, ‘Staff interactions’, ‘Carer’s situation’ and ‘Hospital environment’ and identify suggestions to inform best practice delivery of services with the aim of improving the experience of both people with dementia and their carers.

This analysis identified the importance of well-trained hospital staff, sufficient nursing care and a dignified, person-centred approach in carers’ estimation of hospital care quality. Good communication, involvement and relationship building between staff and carers is key to supporting a good experience for carers and ensuring that the patient’s individual needs are considered when deciding care plans and providing care. Hospital admission can be a significant source of anxiety for carers both regarding its impact on the patient and the consequences for themselves, for example coping with the emotional effects, potential extra responsibilities and managing practical impacts on community care arrangements. Many carers found the hospital environment to be unsuitable for patients with dementia in both practical and social respects.

### Strengths and weaknesses of the study

This is the first systematic review of informal carer perspectives on the delivery of acute hospital care for patients with dementia, and provides the only current synthesis of evidence. The search yielded a large number of results and included both scientific and grey literature. While the researchers had access to the principle healthcare databases, other sources were not accessible and therefore certain publications could have been overlooked. All aspects of the search and analysis were conducted independently by two reviewers followed by in-depth discussions of the material to develop shared understanding. The review was not registered with PROPSERO but robust methodology was used [28].

The qualitative data in the primary studies is specific to its own context and therefore may not be transferrable to other settings. In addition, the limited geographical spread of the papers (UK *n* = 18 and Australia *n* = 2) makes it unlikely that the findings represent a global perspective, and may have limited transferability even within high-income countries due to the variety of healthcare systems. Eight of the selected papers were published by a single research group and reported results of separate analyses from a single RCT. Due to the high quality and level of detail in these papers, their results will have contributed more evidence to this review than other included studies and therefore may have influenced the findings disproportionately. Synthesis required the secondary analysis of qualitative information that had already undergone interpretation in most cases, and therefore could be subject to the influence of different researchers’ methods and perspectives.

This review presents carer perspectives on acute hospital care delivery as a whole, rather than focussing on aspects that are specific only to dementia care because the feedback presented in the primary studies did not separate dementia-specific features of care. We also described a complex interplay between the extracted themes which suggests that there are many factors important in determining carers’ experiences of care and it is difficult to separate the direct contribution of each.

Finally, this review describes the experience and perspectives of informal carers, and therefore may not represent the viewpoint of patients themselves [53]. In order to comprehensively evaluate service delivery in practice, the perspectives of patients and healthcare professionals should also be gathered, completing the triangle of care.

### Relation to similar research

Bridges et al. conducted a systematic review that describes older people’s and relatives’ experiences in acute care settings [54]. This study is not specific to carers’ perspectives or the care of people with dementia but similar themes were identified in this and our study, most notably regarding the importance of relational aspects of care delivery: relationships with staff, a person-centred approach and involvement in decision-making.

The aim of our analysis was to understand the carers’ perspective of the quality of care provided for people with dementia. We sought to identify the key elements of an acute hospital admission that influenced carer views. This differs from the VIPS model of PCC for dementia used in the nursing home setting, which is underpinned by conceptualising personhood through intersubjectivity and social interactions [25]. In the VIPS model, each of the four domains (Value base, Individualised approach, understanding the Perspective, Social psychology) are cross-cutting and focus on the quality and content of social interactions that occur between the person with dementia, healthcare practitioners and carers (as described in the triangle of care model) and recognise the critical role of the carer in providing a valid perspective on the needs and desires of the person with dementia [24–26]. Our analysis is grounded by this theoretical framework and is only valid if the relationship between carers and the person with dementia is intact, if the role of the carer participants is to hold together the delivery of PCC and if the carers share useful insights into the perspectives of the person with dementia. We do not replicate the VIPS and PCC models as we focus on structural elements of care; when social interactions or relational aspects of care are included they are situational and specific to one of the four domains of an admission. We consider that our findings could be used by acute healthcare providers to identify elements of current practice that can be targeted for improvement.

### Implications of research findings

The findings presented in this review highlight aspects of care that could be practically addressed to improve the delivery of care for people with dementia in acute hospitals. Adequate staff training to support understanding of dementia and appropriate care provision is one area of key importance. Creating dementia friendly environments in hospitals has been the subject of increasing attention and guidelines are available to help care providers achieve improvements [22, 55, 56]. Some key recommendations include using clear signage, lighting, colours, pictures and objects to improve orientation and wayfinding, personal items to promote familiarity, and provision of meaningful activity for example walks and outdoor spaces, books, games and memorabilia [55].

Having systems in place to improve communication and involvement of carers would help in providing individualised patient care as well as supporting carers with their own needs. In recognition of the needs of carers, England introduced The Care Act (2014) in April 2015. This gives local councils and NHS bodies a responsibility for assessing carer’s needs and supporting them [57].

The extent of carer involvement will depend on many factors, including whether the patient has the capacity to make decisions about their medical care, and whether the carer has a lasting power of attorney (LPA) for health and welfare. In all situations when a patient lacks capacity it is best practice for the healthcare team to consult with those close to the patient to better understand the patient’s preferences or values, and the carer’s perspective on the proposed action [58]. In the case of an LPA, carers may have to make decisions on the patient’s behalf regarding treatment options and care plans. This activity and potential responsibility can place considerable demands on an individual.

While carers’ individual situations may not be clearly evident to hospital staff, many are under significant strain and have difficulties managing the caring role and its impact on their health: effective provision of information and signposting to services could help address these issues [59].

## Conclusions

This review has identified three aspects of care that influence carer’s perspectives of the quality of a hospital admission for a person with dementia: well-trained hospital staff, sufficient nursing care and a dignified, person-centred approach. Informal carer experience was also more positive when carers were informed, involved and supported during the hospital admission. We suggest that a focus on these factors could improve the perceived quality of a hospital admission by people with dementia and their informal carers.

## Appendix 1

**Table 3:** Medline search strategy

1	(((cognit* or memory* or mental*)adj3(declin* or impair* or los* or deteriorate* or defect* or disorder* or problem*)) or Alzheimer* or dement* or confus*).ti.tb
2	Exp Dementia/
3	Mild Cognitive Impairment/nu
4	Alzheimer Disease/nu
5	1 or 2 or 3 or 4
6	(carer* or caregiv* or care-giver* or spouse-caregiver* or (care* adj2 giv*) or (family adj1 carer*) or family or families or (famil* adj4 member*) or relatives or (informal* adj2 care*) or satisf*).ti,ab.
7	Caregivers/
8	6 or 7
9	(((hospital or acute or medical or patient) adj2 care) or hospital* or acute or hospitali#ation or hospitali#ed).ti,ab.
10	Exp Hospitals/
11	9 or 10
12	((quality or evaluat*) adj4 (healthcare or care or (health adj1 care) or (health adj1 service*) or service*)).ti,ab.
13	Exp “health care quality, access, and evaluation”/
14	12 or 13
15	((satisf* or perspective* or experience* or opinion*) or (survey* or questionnaire* or qualitative* or interview*)).ti,ab.
16	5 and 8 and 11 and 14 and 15

## Appendix 2

### List of organisations’ websites searched for additional publications

1. Alzheimer’s Society (UK): https://www.alzheimers.org.uk/
2. Alzheimer’s Association (US): http://www.alz.org/care/overview.asp
3. Department of Health (England and Wales): https://www.gov.uk/government/organisations/department-of-health
4. Royal College of Nursing (UK): https://www.rcn.org.uk/
5. Age UK: http://www.ageuk.org.uk/
6. Carers UK: http://www.carersuk.org/
7. Alzheimer Europe: http://www.alzheimer-europe.org/
8. Alzheimer’s Disease International: https://www.alz.co.uk/
9. Alzheimer’s Australia: https://www.fightdementia.org.au/
10. World Health Organisation: http://www.who.int/en/
